# SPEED: A Graphical User Interface Software for Processing Eye Tracking Data

**DOI:** 10.3390/neurosci6020035

**Published:** 2025-04-16

**Authors:** Daniele Lozzi, Ilaria Di Pompeo, Martina Marcaccio, Matias Ademaj, Simone Migliore, Giuseppe Curcio

**Affiliations:** 1 A2VI-Lab, Department of Life, Health and Environmental Sciences, University of L’Aquila, 67100 L’Aquila, Italy; daniele.lozzi@univaq.it; 2 Department of Information Engineering, Computer Science and Mathematics, University of L’Aquila, 67100 L’Aquila, Italy; 3 Department of Biotechnological and Applied Clinical Sciences, University of L’Aquila, 67100 L’Aquila, Italy; ilaria.dipompeo@graduate.univaq.it (I.D.P.); martina.marcaccio@graduate.univaq.it (M.M.); matias.ademaj@student.manchester.ac.uk (M.A.); simone.migliore@univaq.it (S.M.); 4 Faculty of Biology, Medicine and Health, University of Manchester, Manchester M13 9PT, UK

**Keywords:** eye tracking, psychometrics, computational psychology, cognitive psychology, moral decision making

## Abstract

Eye tracking is a tool that is widely used in scientific research, enabling the acquisition of precise and detailed data on an individual’s eye movements during interaction with visual stimuli, thus offering a rich source of information on visual perception and associated cognitive processes. In this work, a new software called SPEED (labScoc Processing and Extraction of Eye tracking Data) is presented to process data acquired by Pupil Lab Neon (Pupil Labs, Berlin, Germany). The software is written in Python which helps researchers with the feature extraction step without any coding skills. This work also presents a pilot study in which five healthy subjects were included in research investigating oculomotor correlates during MDMT (Moral Decision-Making Task) and testing possible autonomic predictors of participants’ performance. A statistically significant difference was observed in reaction times and in the number of blinks made during the choice between the conditions of the personal and impersonal dilemma.

## 1. Introduction

Explorations of scenes play a crucial role in psychological research that focuses on attention or visual tasks [1]. Eye-tracking devices are widely regarded as an effective tool for this purpose due to their noninvasive nature, affordability, and ability to provide extensive information. In general, eye-tracking devices offer valuable information on gaze patterns, fixations, and blinks, and some models even allow pupillometry analysis. With the advent of high-speed cameras and infrared light sources, current eye-tracking capabilities include modelling and predicting gaze patterns, calculating fixation durations between saccades and microsaccades, blink and half-blink frequencies, and pupillometry [2]. Since the latter half of the twentieth century, scientists have developed several methods [3], both invasive and non-invasive, to record eye movements using mechanical and photographic techniques. Although traditionally recorded with contact lenses with mirrors, eye movements captured by camera-based devices have allowed for non-intrusive methods of eye tracking. Eye trackers are now widely used in scientific research, enabling the acquisition of precise and detailed data on an individual’s eye movements during interaction with visual stimuli, thus providing ample information on visual perception [4] and associated cognitive processes [5], particularly in the areas of emotional and cognitive evaluation.

### 1.1. Applications of Eye Tracking

These basic capabilities of eye tracking have paved the way for a wide range of applications in psychological research. Eye tracking is therefore useful for studying cognitive processes, particularly in the areas of emotional and cognitive evaluation. The sphere of emotions can be studied using eye tracking, analysing changes in pupil size, blink frequency, and gaze patterns [6]. Pupil dilation has been associated with increased emotional engagement, arousal, and cognitive effort [7], with lower blink frequency indicating increased attention, arousal, sensory processing, and heightened cognitive load [8,9,10]. In addition, changes in gaze patterns towards emotionally salient stimuli may provide insight into emotional responses and engagement with the environment [1].The study of eye responses to mental imagery and recollection is of great interest to researchers, as this can provide insights into the role of eye movements in memory recall [11]. Applications of eye-tracking devices in psychological research also involve visual search tasks to investigate object detection and information processing during scene viewing, both in simulated and natural environments [2]. The variables measured by the eye tracker (described above) are also useful in the induction of emotions, as they provide support for subjective measures in confirming and demonstrating the effectiveness of induced emotions [12]. Gaze-avoidance behaviours have also made eye tracking a promising tool in the diagnosis of mood and neurodevelopmental disorders such as social anxiety disorder and autism [13,14]. In [15], an eye-tracking system for diagnosing children with Autism Spectrum Disorder (ASD) was developed by revealing differences in visual fixation patterns between neurotypical individuals and those with high-functioning ASD during the visual processing of facial features. Individuals with ASD tended to fixate less frequently on key facial regions, such as the eyes and nose [16]. The system also provides precise data on the mechanisms and time course of cognitive processing, offering advantages over traditional behavioural measures. It provides researchers with a way to observe and analyse how individuals visually process information, make decisions, and allocate attention during cognitive tasks. By assessing the position of a person’s gaze in milliseconds, eye-tracking technology offers detailed and accurate data on the mechanisms and time course of cognitive processing. It has also led to important advances in the understanding of the reading process, shedding light on the mechanisms and processes involved in reading [17]. In the context of text comprehension, eye tracking provides a continuous, real-time record of reading performance, allowing researchers to follow eye movements as participants interact with text. This allows moment-by-moment processing demands to be captured during reading. The duration of fixation, the number of fixations, and the number of regressions reflect the cognitive processes involved in text comprehension, such as the difficulty in processing specific words or phrases, the need for multiple fixations on challenging text segments, and the sequence in which readers process the text [18]. Regarding decision-making processes, the eye tracker provides a better understanding of how people process information, form preferences, and make decisions in different contexts. Changes in eye movement patterns can be a consequence of decision making. People tend to look more frequently at options they have decided are better, suggesting that eye movements may reflect preferences and influence final choices [17]. It has been suggested that gaze may play an active role in the decision-making process, influencing the choices made. It emerges that the gaze is directed towards the next chosen option, indicating a dynamic interaction between eye movements and decision making [19,20]. Eye tracking is also used in neuromarketing, a field of study that uses neuroscientific methods to analyse and understand human behaviour, particularly as related to marketing. This approach is based on the use of techniques to study the brain responses of individuals exposed to marketing stimuli to better understand consumer motivations and decision-making processes. The aim is to obtain more in-depth information on consumers’ emotional and cognitive reactions [21]. It can provide information on consumers’ intention to purchase products, as well as on the unconscious factors that influence their choices and preferences [22]. Eye tracking is also promising in the study and diagnosis of autism.  The authors in [15] developed an eye-tracking system for children with ASD. The main purpose was to analyse and understand the specific visual attention patterns of this population, thus providing a tool for accurate data collection and early diagnosis. The use of eye-tracking technology has revealed significant differences in visual fixation patterns between individuals with high-functioning autism and neurotypical individuals during the processing of social information. Individuals with autism tend to fixate less frequently on key facial regions, such as the eyes and nose [16].

### 1.2. Eye-Tracking Devices

A wide range of eye-tracking equipment is available on the market, designed to serve both scientific and marketing research. Among the available products, Tobii Pro eyetrackers, particularly the Spectrum series and the wearable Glasses 3, are characterised by their versatility and accuracy, which make them suitable for both controlled laboratory contexts and more operative and contextualised environments. The EyeLink series, including Portable Duo and 1000 Plus/2000 Plus, is determined as a premium option for scientific research focusing on high methodological rigor, as they have high accuracy and sample rates. In addition, the Gazepoint product line, GP3 HD and GP3, provides affordable and reliable options for a broad variety of eye tracking applications. Pupil Labs hardware, like Pupil Core and Pupil Invisible, are one of a kind in their portable form factor and in being capable of recording in difficult and ecological settings.Pupil Labs [23] (https://pupil-labs.com/) is an open source eye tracking solution with applications in various research fields, particularly cognitive neuroscience. The Neon eye-tracking module (Pupil Labs, Berlin, Germany) provides a cheaper mobile alternative to high-end remote eye-tracking systems such as EyeLink 1000 (SR research), while retaining extensive research usability [24]. Neon utilises a mobile phone application interface to capture recording data and upload them to an online database, termed Pupil Cloud, for data organisation, analysis, and visualisation. The data processing platforms currently available on the web-based Pupil Cloud include gaze mapping onto a static surface (Marker Mapper); mapping gaze onto a reference image within a moving recording in 3D space (Reference Image Mapper); and detecting gazing at facial features (Face Mapper). In the first two types of mapping functions, a Region of Interest (ROI) can be bounded either manually in the Pupil Cloud or automatically by utilising Alpha Labs (https://docs.pupil-labs.com/alpha-lab/gaze-metrics-in-aois/) in order to gain insights on the fixation duration and frequency between separate items. The Face Mapper function is used to work out which components of the face the subject focuses on. Detections involve the definition of a rectangle that delimits the face, which makes it possible to understand when and where faces are visible to an individual. This allows the automatic mapping of gaze data on faces and calculates the location of key facial landmarks in the image for everyone. It is very useful in studies investigating social functioning in autism. Furthermore, the eye tracker was also used in a study to investigate how aesthetic perception abilities can influence social functioning and social relationships in people with autism. The instrument made it possible to verify that people with ASD show differences in fixation patterns during the aesthetic perception task compared to neurotypical subjects [25]. In conclusion, the eye tracker is a versatile tool with applications in a wide range of research fields. For this reason, there must be more understanding of the data that can be extracted and analysed using this tool. Pupil Labs has developed cutting-edge eye-tracking hardware and software, enabling the visualisation and utilisation of hidden patterns in human behaviour. It also offers a cloud (Pupil Cloud) in which the data are uploaded through their official applications and preprocessed according to user goals (such as Heat Maps and Area of Interest Maps). As the adoption of eye-tracking technology grows, so does the need for accessible tools to analyse the structured data output of platforms like Pupil Cloud. This data structure has peculiarities caused by the instruments that require suitable software or adaptations, as there is currently no standard for eye tracker data, contrary to what is carried out, for instance, in medical imaging [26,27]. This paper presents the first version of our software, specifically created to help researchers—even those without programming experience—extract key features from Pupil Cloud’s data, built for its specific data structure, and visualise them in a way that is directly applicable to psychological research questions.

## 2. Eye-Tracker Software

Many online libraries allow for the capture of gaze patterns via webcam (https://github. com/kongmunist/Webcam-Eyetracking) and for eye tracking data analysis (https:// github.com/topics/eye-tracking), but none of them are suitable for researchers without any programming knowledge. Available libraries [28,29] allow for no-cost eye tracking and data analysis, but the inability to handle eye tracker-specific datasets without prerequisite coding knowledge renders it incompatible for the average user of a Pupil Labs Neon device. The landscape of Python/R libraries for eye tracking research encompasses a wide range of experimental needs with extensive analysis capabilities through scripting. PyGaze [30] is a high-level toolbox that allows for the design of visual and auditory stimulus presentation and response collection through various input devices. It requires minimal effort from the programmer while maintaining flexibility in its functionality. PsychoPy [31,32] enables the creation of projects for psychophysiological experimental research by integrating eye-tracking functionalities within broader behavioural research. Researchers can effectively write scripts for eye-tracking experiments using established hardware configurations such as EyeLink, Tobii, and Pupil Core trackers, making it versatile for various experimental setups. This integrative capability enhances the overall experimental workflow, classifying PsychoPy as central to visual processing studies employing eye-tracking technologies. GazeParser [28] provides an open source and cross-platform solution for low-cost eye tracking and analysis. GazeParser allows researchers to start and stop data recording with precise timing, thus ensuring the accuracy and reliability of the collected eye movement data. The synergy of this library with other Python-based experimental control systems like PsychoPy amplifies its functional scope, enabling sophisticated data collection methodologies that appeal to behavioural researchers. pyeTribe [33] is a software package specifically designed for interactive tasks in studies involving economic games, offering simultaneous calibration and data collection across multiple eye trackers, which optimises the utility of low-cost eye tracking solutions. This multi-participant recording capability is crucial in modern experimental designs that require real-time data from several subjects. The Pyff library [34] has the advantage of being able to program neuroscience experiments while also providing feedback to the participant. It features various base classes for common feedback mechanisms along with support for external hardware interactions, enhancing the overall utility of eye tracking applications in experimental paradigms. The MNE-Python library [35] allows for the analysis of multimodal M/EEG and eye tracking data. TobiiGlassesPySuite is a software that allows for the extended exploitation of the Tobii Pro Glasses 2 [36], making improvements on the already available Tobii-provided software. The analyses utilised are especially useful for studying visual attention processes, especially in activities such as gamification on Over-The-Top (OTT) platforms [37]. It is also extensively utilised in various domains like video applications, where visual analytics tools are used for sensemaking and cluster analysis [38]. Additionally, software tools such as the ELAN Analysis Companion (EAC) assist in the temporal analysis of gaze-tracking data and the enhancement of eye tracking datasets [39]. The Eyetrace software suite [40] offers various evaluation techniques that are compatible with multiple eye-tracker models, thus overcoming the obstacle of data standardisation across eye trackers. Pupil Labs offers an integrative solution for eye-tracking devices, allowing for gaze capture and simple data analysis via their website. Pupil Cloud (https://cloud.pupil-labs.com/) currently offers a limited number of data analyses and lacks a data processing function, rendering eye tracking data difficult to manipulate for novices of eye tracking research. The aim of this work is to present labScoc Processing and Extraction of Eye tracking Data (SPEED), a Graphical User Interface (GUI) software for the data analysis of raw data obtained from Pupil Lab Neon modules and downloadable from Pupil Cloud [41]. The main advantages of SPEED reside in its specific compatibility and adaptation to the data structure generated by the Pupil Lab Neon system and its ease of use for users without specific programming skills, allowing them to obtain summary statistical analyses.

## 3. SPEED Software

Pupil Cloud offers many methods to extract information by preprocessing the raw data acquired by the Neon device [42]. However, Pupil Cloud operates as an online platform and, while processing the data, does not directly provide the summary statistical outputs that researchers often require. To bridge this gap, we present software written in Python (version 3.13.0) [43] called SPEED. SPEED is specifically designed to calculate and export these summary statistics, starting from the raw, preprocessed data obtained from Pupil Cloud, thus helping researchers with the subsequent feature extraction and plotting steps. The software offers a GUI to make it easy to use for researchers without any knowledge of coding. This software is designed to improve the analysis pipeline for data acquired with Neon and can also extract information after applying enrichment methods, such as “Marker Mapper” or “Reference Image Mapper”, if the corresponding enrichment file is provided.

### 3.1. Events Loader

In the initial window, once the user has chosen the directory containing the files related to blinking, gazing, fixations, pupillometry, saccades, enrichment, and events, they have the option to import an additional event file in a CSV format. This new file can replace the events in the original data obtained from the cloud. This functionality enables the user to generate epochs within the eye tracking dataset if another device is involved.

### 3.2. Features

For SPEED to function, a ROI, defining the field of observational interest detected by the outward-facing cameras of the Neon module, is required. The ROI eye tracking is carried out as follows:

- Placing April Tags on each corner of the observation area (https://github.com/April Robotics/apriltag) and performing a “Marker Mapper” Enrichment
- Providing the reference image and scanning and recording for the “Reference Image Mapper” (https://docs.pupil-labs.com/neon/pupil-cloud/enrichments/).

After the enrichments are completed, enrichment data on the downloads tab of the Pupil Cloud workspace and its files can be placed in the same directory as the recording data. In the first step of data processing, if one of the above methods of ROI definition is placed in the same folder, SPEED adds a binary feature called “detected on ROI” that adds information to the blink, pupillometry, and saccade files based on gaze and fixation data; registered data points are those occurring when the gaze is within the ROI, meaning that for each frame of the recording, SPEED assigns a binary value, 1 or 0, depending on whether the gaze was detected within the ROI or not, respectively. For pupillometry data, this binary value was given as True or False. For each event, the software computes the same features. If there are no events (the files are always needed but only with start and end events), the software computes each data analysis feature for the entirety of the recording. The following section explains the features extracted by SPEED with their theoretical references, and in Table 1, the features extracted by SPEED are listed. For this first version of SPEED, the use of enrichment is required. The future version will also include the data structure without enrichments.

The algorithms written to extract information from or add information to the data downloaded from Pupil Cloud are reported in Appendix A. Algorithms A1 and A2 are used with gaze data to extract the movement between fixations; Algorithms A3–A6 are used inside other algorithms as callable functions. Algorithms A7 and A8 are used to create lists of gazes related to fixations and gazes related to movements.

**Figure 1:**
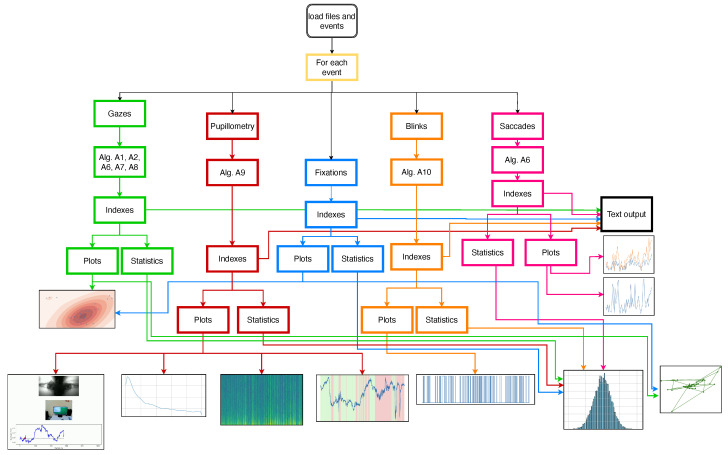
This flowchart illustrates the analysis procedure for eye tracking data via custom software. The software analyses fixations, gaze, pupillometry, and blink data to generate and display various indices, statistics, and plots. Algorithms are detailed in the appendix. The colours depict features and their preprocessing flows. The outputs include image, video, or text and may overlap for certain features.

The flow chart shown in Figure 1 explains how the SPEED works and the pipeline of the pre-processing stage, the feature extraction stage, and the plot stage.

Gazes and fixations are the values normalised and extracted from the table created while enriching the enrichment on the relevant Pupil Cloud workspace. In Table 2, the equations used in SPEED are reported.

In Figure 1, the flowchart represents how the software works. In summary, SPEED processes raw data related to gaze, pupillometry, and blinks to generate various indices, statistics, and visualisations. The process begins with loading eye tracking data files and specifying events of interest. The software can handle data from single or multiple participants and can process multiple events within each data file. The software then performs data enrichment by processing the raw gaze, pupillometry, and blink data, applying algorithms to identify fixations, saccades, and other relevant eye movement metrics. Based on the enriched data, the software calculates various indices, which are likely to quantify aspects of visual attention, such as the duration of the fixation, the amplitude of the saccade, and the dilation of the pupil. Statistical analyses are then performed on the computed indices, which can involve comparing the indices between different events, time points, or groups of participants. Finally, the software generates plots to visualise the data analysed, including heat maps, gaze plots, and time-series graphs. The output of the software is a text file containing the computed indices, statistical results, and visualisations, which can be used to draw conclusions about visual attention and eye movement behaviour.

### 3.3. Plots

**Figure 2:**
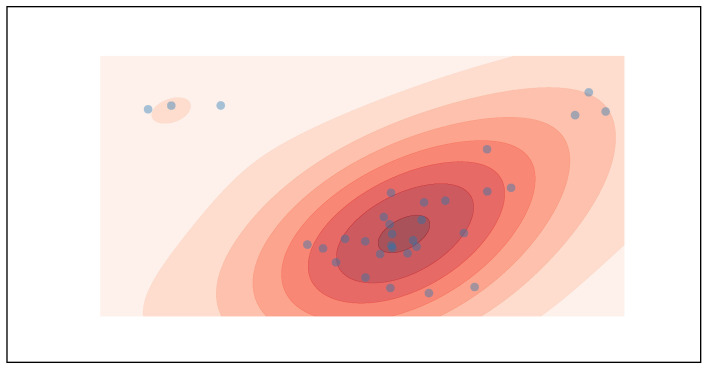
The cloud point of fixations shown after the preprocessing. This plot could also be produced using gazes. This image qualitatively indicates the most observed mean position together with its associated distribution on the ROI.

The SPEED software offers a range of graphical visualisations for pupillometry time series. It allows for the creation of plots in which colours differentiate events according to whether subjects are looking inside (green) or outside (red) a ROI. Beyond this, SPEED can generate a cloud point visualisation, as shown in Figure 2, which overlays fixation points to highlight areas of concentrated gaze.

**Figure 3:**
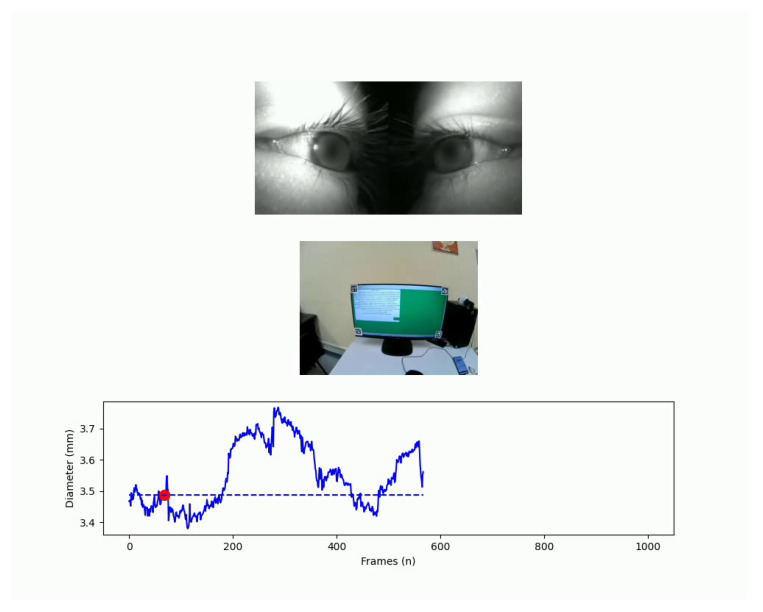
A frame of one output of SPEED. This output includes external and internal videos and the pupillometry amplitude time-series. The pupils are blurred to preserve the biometric data of the participant.

This visualisation includes Gaussian kernel density estimation to show probability density and can use a user-selected image as a background to provide context to the areas of interest. Another functionality is video pupillometry, which, as shown in Figure 3, generates a synchronised display of the pupillometry time series, a real-world video feed, and an eye video, helping to understand the real-world factors influencing changes in pupil size.

**Figure 4:**
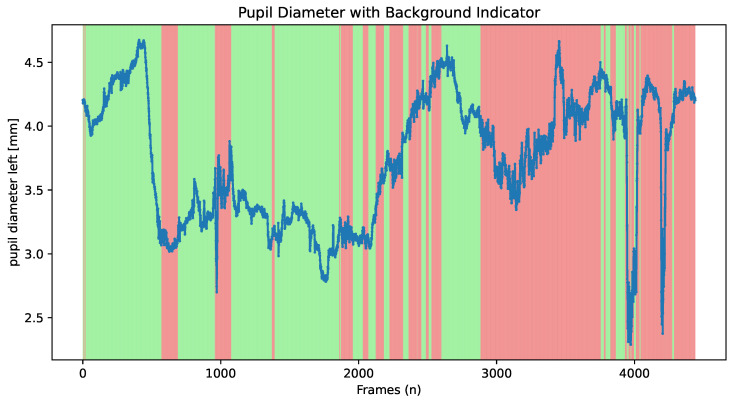
Plot of the pupillometry data, including details on when the subjects focus on the surface. The drops represents the blink, which is useful to analyse if it occurs in a specific period. When it is looking at the ROI, it is shown in green; when it is not, it is in red.

Furthermore, the software provides a feature, illustrated in Figure 4, to plot pupillometry time series with colour coding to indicate when a user is looking at the screen, which is useful to analyse focus within the ROI.

**Figure 5:**
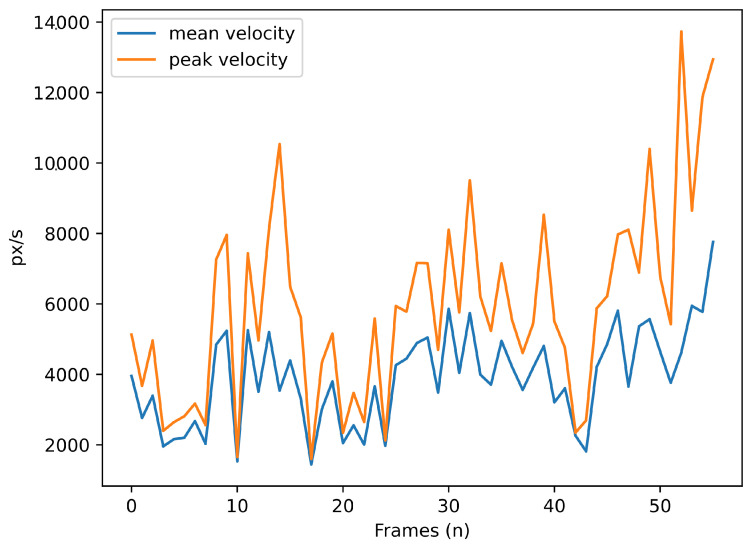
Mean (in blue) and peak (in orange) of saccade velocity (pixels/ms) over time (frames).

**Figure 6:**
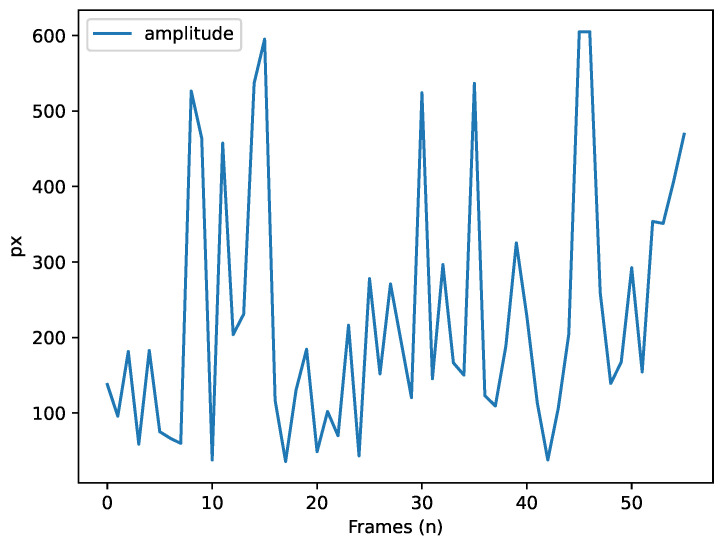
Saccade amplitude (pixels) over time (frames).

**Figure 7:**
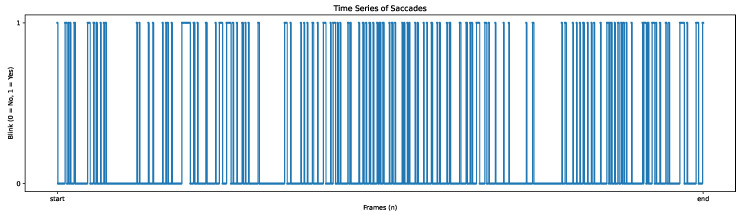
The blink time series visualised. The values on the y-axis are binary (1 when blinks occurs).

For the saccade analysis, SPEED allows users to visualise the mean and maximum velocity of saccades (Figure 5) and the amplitude time series (Figure 6), providing insights into the characteristics of eye movement. Blink events are also visualised as discrete time series, where a value of 1 represents a blink, as shown in Figure 7, offering an overview of blinks during tasks; this process is further enriched by Algorithm A10 to determine if blinks occur within or outside the ROI.

**Figure 8:**
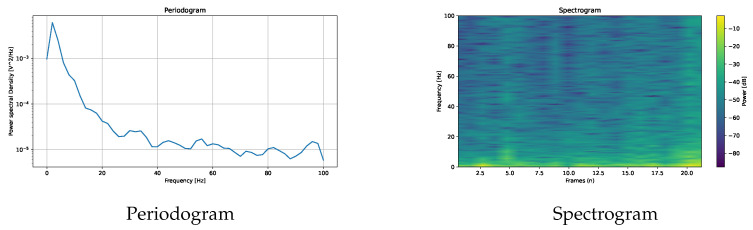
Two plots based on Fast Fourier Transform.

**Figure 9:**
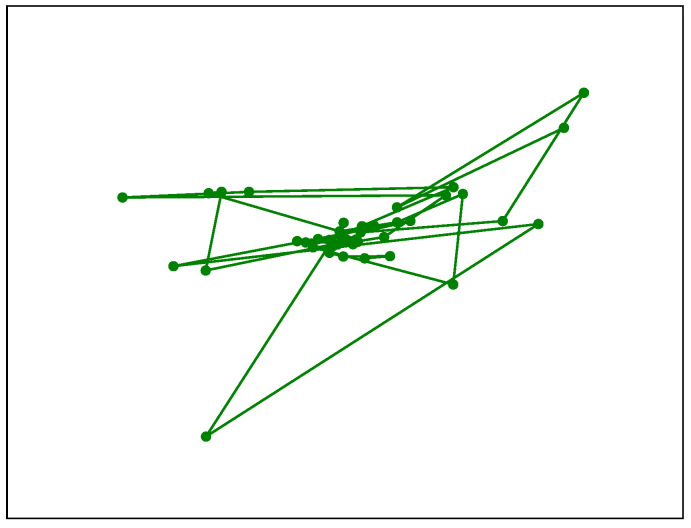
Plot of the movements (fixations or gazes). This image qualitatively indicates the path of the fixations and gazes on the ROI.

In addition to time-series plots, SPEED supports frequency domain analysis through Fourier transforms, visualising both periodograms and spectrograms using Welch’s method, as shown in Figure 8, to analyse the signal frequency and the power spectral density. Finally, movement data extracted using Algorithm A2 can be represented as a path graph, exemplified in Figure 9, to visualise gaze or fixation movements. Moreover, some features are available in histogram form, as shown in Figure 1.

### 3.4. Interface

**Figure 10:**
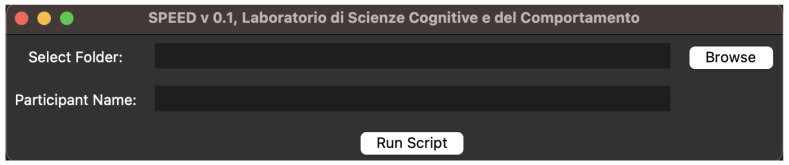
The first input field, *Select Folder*, is first the folder path, and the second input field, *Participant Name*, is for the name or code of the participant that will be used to rename the output.

The interface is designed to be extremely simple. It allows the loading of two pieces of information: (1) the folder in which the files downloaded by the Pupil Cloud are present and (2) the name (or code) to assign to the participant. In Figure 10, the interface of the first alpha version of SPEED is shown. In the selected folder, all files need to be loaded.

## 4. Example of Use

In this section, a simple use case of this software is shown in combination with PsychoPy [31]. The purpose of this preliminary study is to evaluate the oculomotor correlates of a Moral Decision-Making Task (MDMT) and test possible autonomic predictors of participant performance. The objective is to demonstrate the utilisation of the software and to highlight its potential application within the realm of cognitive data analysis.

### 4.1. Background

Decision making is one of the highest and most complex cognitive skills. In particular, moral dilemmas are used to investigate the interplay between emotional and cognitive processes in moral judgement and decision making [44]. During visual exploration, our eyes move both in order to capture new information (bottom-up processing) and in response to pre-acquired information (top-down processing). Although bottom-up processing promotes more explorative, stimulus-driven gaze during information collection, eye movements during higher cognitive functions such as information contextualisation and choice deliberation are determined more so by both stimulus-relevant and endogenous top-down processing factors, such as interpreting facial features and moral judgment [45,46]. Eye movements, and therefore decision making itself, can also be influenced by emotional states, such as stress or task-relevant pressures [47]. Eye movements and visual exploration are used in various fields; it is known that whenever we must decide, we mentally represent the problem and then consider the options, examining them before making a final decision. Eye movements serve as a rich source of information for understanding the cognitive processes underlying decision making [19]. While we reason about the most convenient choice, the posterior parietal cortex, frontal ocular field, and motor cortex intensify their activity [48]. Some of these brain areas project to the superior colliculus, which in turn coordinates eye movement. As we look at the options, eye movements change (e.g., speed and force of the saccades) and can provide us with real-time patterns of thought processes involved in decision making [49]. Although requiring several layers of data analysis to unobscure [45], the resulting data from eye movements can be analysed to provide us with real-time insights into the cognitive processes involved in decision making [49]. Technological applications of this knowledge could also include the development of choice-predicting eye-tracking software for a wide range of services.

### 4.2. Participants

This pilot study involved five healthy volunteers, all of whom were native Italian speakers. All were subjected to a Moral Decision-Making Task (MDMT) task while being made to wear a Neon model eye tracker (PupilLabs GmbH (https://pupil-labs.com/products/neon); Berlin, Germany). All individuals were subjected to the same protocol, with male and female respondents being presented with a male or female-voiced protocol, respectively.

### 4.3. Moral Decision-Making Task

A MDMT consisting of 56 dilemmas was used, originally published by Green on the investigation of emotional engagement in moral judgment [48] and translated to Italian [50]. The set of dilemmas was divided into three main conditions in which the level of involvement in the participant’s moral choice changed:

- Eight non-moral (NM) or control dilemmas, in which there is no moral choice (no emotional involvement);
- Eight impersonal moral (MI) dilemmas, in which the protagonist neither causes nor induces harm to others by his or her actions but behaves in a socially wrong way (significant emotional involvement);
- Eight personal moral (MP) dilemmas, in which the protagonist behaves in ways that may induce harm to others but with good and positive purposes (very high emotional involvement).

Then, three variations of the MP and MI dilemmas were created containing audio-visual differences:

- Unobserved (U): the dilemma was kept unedited, with no observers present, plus the additional audio cue “Sai che nessun altro ti vede” (*Translation*: “You know that no one else is observing you”);
- Media (M): a dilemma where journalists or members of the media observe the experimental subject, plus the additional audio cue “Sai che sei osservato da un giornalista” (*Translation*: “You know that you are observed by a journalist”);
- Authority (A): a dilemma in which a law enforcement officer or security guard observes the subject, plus the additional audio cue “Sai che sei osservato da un poliziotto” (*Translation*: “You know that you are observed by a policeman”).

In summary, the NM condition had eight dilemmas, whereas the MI and MP conditions had twenty-four dilemmas, eight with “Unobserved” variation, eight for “Media”, and eight for “Authority” variations. In Table 3, a summary of the conditions, variations, and quantity is shown.

All dilemmas were created through audiovisual stimuli, in which the image depicted the situation and the audio described it, finally posing the question of how to make the moral choice. In addition, to reduce bias, the voice reading of the questions was created using artificial intelligence services (https://elevenlabs.io/), creating both male and female versions. The male version “George” was used with male participants, while the female version “Dorothy” was used with female participants. Finally, the paradigm was programmed using PsychoPy [31]. After every 7 dilemmas, there was a short 30-second pause, with a longer break of a subject-chosen time frame after 28 dilemmas.

### 4.4. Procedure

Before starting MDMT, each subject provided his informed consent and was verbally instructed about the task to be performed. The experiment took place on an intel i5 PC with a 23″ monitor (20.05 × 11.28 in) placed 75 cm away from the subject’s eyes when seated. The top of the monitor was positioned at eye level, and the subject was instructed to keep their head still as much as possible when responding to dilemmas. The offset gaze calibration was performed by instructing the individual to fixate on a central point of the monitor.

**Figure 11:**
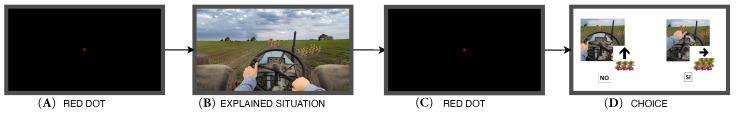
Experimental paradigm. The sequence of dilemma presentation: (**A**) an inter-stimulus interval (ISI), displayed for 1-s; (**B**) the main stimulus of the dilemma, presented for the entirety of the related audio stimulus (27.8 ± 3.00 s, Mean ± Standard deviation); (**C**) a second ISI, 1 s; (**D**) and the choice stimulus of the relevant dilemma, 4.34 ± 2.01 s (mean ± st. dev.). The loop is repeated for 56 dilemmas with either a 30-s break after every 7th dilemma or a participant-resumed pause after the 28th.

**Figure 12:**
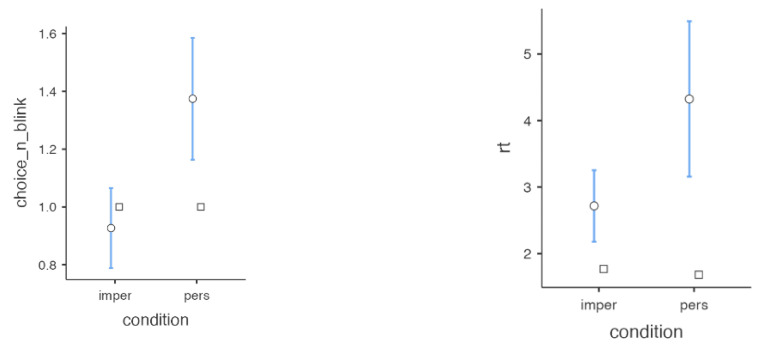
Results of the MDMT experimental paradigm for decision-making. The variable “choice_n_blink” represents the number of blinks computed using an adapted version of SPEED for the two conditions ‘ìmpersonal” and “personal”, and “rt” indicates the reaction time. The circle represents the mean and the square represents the median. In blue, the range of the values is shown.

Next, the experimenter helped each subject to wear the eye tracker correctly, performing the calibration of the instrument to properly collect data. Each person was trained to look at a short set of dilemma questions and choose among the various options. The experimental paradigm is shown in Figure 11. Five participants were recorded, and the results are shown in Figure 12.

### 4.5. Data Analysis

Descriptive statistics were performed on age and gender, as reported in the Participants Section. To test for possible autonomic predictors of participants’ performance, we conducted a comparison of different conditions (personal dilemmas VS impersonal) using an independent samples *t*-test on all eye-tracker measurable variables (fixations, saccades, gaze path, gaze duration, pupil size, blink rate) and with the MDMT (responses and reaction times). All analyses were performed using Jamovi software [51]. Participants who exhibited more than five blinks were omitted due to data acquisition errors; the average duration of the “choice” was approximately 3.5 s. Given that a typical blink rate within this time frame is approximately 15–20 blinks per minute [52], the error threshold was established at fewer than five blinks during the choice audio.

### 4.6. Results

A statistically significant difference was observed in the reaction times (t = −4, p=0.001) and in the number of blinks made during the choice (t = −3.3, p=0.001) between the personal and impersonal dilemma conditions. Subjects on average took longer and made more blinks in making decisions when the choices to be made involved moral dilemmas with a high level of participation. Longer reaction times and a higher frequency of blinks when making personal moral decisions than when making impersonal ones indicate increased attention, intensity of emotional responses, and cognitive load [9].

## 5. Conclusions

The objective of this work was to introduce the SPEED software along with a specific application scenario. SPEED was designed to help researchers who lack programming experience but use the Neon Pupil Lab device, enabling them to quickly process and visualise the data collected from this device. It accepts enrichment files from the Pupil Cloud platform as input and allows for the analysis of individual events. Additionally, SPEED provides certain details and preliminary statistics that are not directly offered by Pupil Cloud/Alpha Lab (https://docs.pupil-labs.com/alpha-lab) or Neon Player (https://docs.pupil-labs.com/neon/neon-player/). A significant current limitation is the lack of available tools that both accept the specific data structure generated by Pupil Cloud as input and do not require coding skills from the user. This makes it difficult to directly compare similar and code-free tools. Secondly, the preliminary results presented in this study to show SPEED’s functionality are based on a small sample of participants, but the primary aim was to demonstrate an application of SPEED without any inference on the results. Therefore, while this sample size is not suitable for broad generalisations, it was deemed sufficient for illustrating the software’s use case. Future enhancements will focus on adding new features and refining the graphical user interface to make it more accessible to researchers.

## Figures and Tables

**Table 1:** Summarised eye-tracking features.

Category	Algorithm(s)	Indexes	Plots	Statistics
Blink	Adding “On the surface”	Count Duration (total, average, and surface)	Time-series Locations (ROI)	Duration histogram (fixations)
Fixations		Count Duration (total, average, max/min) Position (max duration, average) Distance (absolute/relative: total, average)	Cloud points Absolute path Relative path	Duration histogram
Gaze	Indexing movements Extracting movements	Count (total, per fixation, per movement) Movement duration (total, average) Distance (absolute/relative: total, average, max/min)	Cloud points (per fixation) Absolute/relative path (per movement)	Distance histograms (absolute/relative, gaze/movement)
Saccade		Count Duration (total, average, max/min) Amplitude (average, max/min) Speed (average, max/min, peak)	Amplitude Mean and peak velocity	Duration histogram
Pupillometry	Adding “On the surface”	Diameter (first/last, max/min, average)	Video + diameter Spectrogram Periodogram ROI pupillometry	Diameter histogram

**Table 2:** Equations.

Equation	Description
$euclidian\_distance=\sqrt{{\left(x_{2}-x_{1}\right)}^{2}+{\left(y_{2}-y_{1}\right)}^{2}}$	Calculates the Euclidean distance between two points, $\left(x_{1},y_{1}\right)$ and $\left(x_{2},y_{2}\right)$, in a two-dimensional plane.
$element_{max}=\max\left(list\right)$	Finds the maximum element within a given list.
$element_{min}=\min\left(list\right)$	Finds the minimum element within a given list.
$list_{sum}=\sum_{i=1}^{list_{i}}list_{i}$	Calculates the sum of all elements in a given list.
$list_{length}=\mathrm{length}\left(list\right)$	Determines the number of elements (length) of a given list.
$list_{avg}=\frac{\sum_{i=1}^{list_{i}}list_{i}}{list_{length}}$	Computes the average (mean) of the elements in a given list.
$list_{\sigma }=\sqrt{\frac{1}{list_{length}}\sum_{i=1}^{list_{length}}{\left(list_{i}-list_{avg}\right)}^{2}}$	Calculates the standard deviation of the elements in a given list, indicating the dispersion of the values around the mean.

**Table 3:** Conditions and variations of this experimental paradigm.

Condition	Variation	No.
Non-Moral (NM)	-	8
Personal (MP)	Unobserved (U-MP)	8
	Authority (A-MP)	8
	Media (M-MP)	8
Impersonal (MI)	Unobserved (U-MI)	8
	Authority (A-MI)	8
	Media (M-MI)	8
Total		56

## Data Availability

The presented software is available on Zenodo at the following link: https://doi.org/10.5281/zenodo.14049987. The data generated and analysed during the current study are not publicly available due to the fact that this study focuses on the presentation of SPEED, which are used as examples of a pilot project, but are available from the corresponding author on reasonable request. The modified code for the data analysis that interacts with the PsychoPy output are available on Zenodo at the following link from the corresponding author under user request. The stimuli and PsychoPy project that support the findings of this study are available on Zenodo at the following link from the corresponding author under user request. The code is also hosted on GitHub (SPEED: https://github.com/dani elelozzi/SPEED).

## Abbreviations

The following abbreviations are used in this manuscript:

ASD	Autism Spectrum Disorder
GUI	Graphical User Interface
MDMT	Moral Decision-Making Task
ROI	Region of Interest
SPEED	labScoc Processing and Extraction of Eye Tracking Data

## Appendix A. Algorithms

### Appendix A.1. Indexing Movements

Algorithm A1 accepts as input a table of gaze data to identify and index non-fixation eye movements. Initially, the subfunction FillMissingFixationID cleans the data by replacing non-numeric fixation_id entries with a standard sentinel value −1. This is carried out because when there is a numeric value, it is associated with a fixation and not a movement. Next, the main function IndexMovement iterates the table, using these −1 markers to identify sequences corresponding to non-fixation events, and then assigns a unique index to each identified movement sequence. The algorithm returns the modified table, which now contains both the original fixation IDs and these new indices representing the movements between fixations.

**Algorithm A1** Indexing movements
1: **function** FillMissingFixationID(TABLE gaze_mark) 2: **for** fixation_idinTABLE gaze_mark **do** 3: **if** fixation_id is not a number **then** 4: fixation_id=−1 5: **end if** 6: **end for** 7: **return** TABLE gaze_mark 8:** end function** 9: 10:** function** IndexMovement(gaze_mark) 11: *TABLE gaze_mark* ← FillMissingFixationID(*TABLE gaze_mark*) 12: variablen_movement=0 13: **for** fixation_id in TABLE gaze_mark **do** 14: **if** fixation_id == −1 **then** 15: n_gaze = row number of fixation_id 16: **while** (fixation_id≠−1) **do** 17: fixation_id = row number of fixation_id + 1 + 0.5 18: n_gaze=n_gaze+1 19: **end while** 20: **end if** 21: **end for** 22: **return** *TABLE gaze_mark* 23: **end function**

### Appendix A.2. Extracting Movements

Algorithm A2 describes the procedure for extracting the quantitative metrics characterising eye movements from gaze data. It accepts as input a table of movement data, such as saccades, identified by non-integer values of fixation_id. The algorithm iterates through the data table to find these segments and, for each identified movement, determines the initial and final coordinates by relying on auxiliary functions (GetFirstElement, GetLastElement). It then calculates two separate metrics: “actual motion”, defined as the direct Euclidean distance from the starting point to the end point of the segment, and “absolute motion”, calculated as the sum of the Euclidean distances between all consecutive path points within the segment. These values of actual and absolute motion are collected for each identified segment and returned in two separate lists as the output of the algorithm.

**Algorithm A2** Extracting Movements
1: **function** ExtractMovements(gaze_mark) 2: *mov_eff_list* ← empty list 3: *mov_abs_list* ← empty list 4: **for** row in *TABLE gaze_mark* **do** 5: **if** row.fixation_id≠integer **then** 6: start_x,start_y← GetFirstElement(row.x, row.y) 7: path_list← empty list 8: **while** Type(row.fixation_id) ≠ integer **do** 9: x1, y1 = row-1.x, row-1.y ▹ Previous row 10: x2, y2 = row.x, row.y ▹ Current row 11: movement = euclidean_distance(x1, y1, x2, y2) 12: path_list.append(movement) 13: row=row+1 14: **end while** 15: end_x, end_y← GetLastElement(fixation_id) 16: effective_movement←euclidean_distance(start_x,start_y,end_x,end_y)) 17: mov_eff_list.append(effective_movement) 18: mov_abs_list.append(sum(path_list)) 19: **end if** 20: **end for** 21: **return** mov_eff_list, mov_abs_list 22: **end function**

### Appendix A.3. Get the First Element of a List

Algorithm A3 presents the GetFirstElement support function. This utility takes an input list (inputList) and directly returns its first element, which is located at index 0. It is utilised by Algorithm A2 to access the starting point of a data sequence.

**Algorithm A3** Get the First Element of a List
1: **function** GetFirstElement(inputList) 2: **return** inputList[0] 3: **end function**

### Appendix A.4. Get the Last Element of a List

Algorithm A4 details the GetLastElement support function. This utility takes an input list (inputList) and returns its final element. It achieves this by accessing the element at index −1, based on a common indexing convention for retrieving the last item in sequence-based data structures. This function is used within Algorithm A2 to obtain the terminal point of a data sequence.

**Algorithm A4** Get the Last Element of a List
1: **function** GetLastElement(inputList) 2: **return** inputList[−1] 3: **end function**

### Appendix A.5. Euclidian Distance

Algorithm A5 describes the AbsoluteDistance procedure to calculate the direct Euclidean distance between the start and end points of a sequence stored in a list. Assuming inputList contains coordinate data (x, y), it first obtains the starting coordinate using the GetFirstElement supporting function (detailed in Algorithm A3) and the final coordinate using GetLastElement (Algorithm A4). Then, it computes and returns the standard Euclidean distance between these two specific points (firstElement and lastElement), representing the separation between the start and the end of the path defined by the input list.

**Algorithm A5** Euclidian distance from last and first element on a list
1: **procedure** AbsoluteDistance(inputList) 2: firstElement←GetFirstElement*(inputList)* 3: lastElement←GetLastElement*(inputList)* 4: **return** EuclideanDistance(firstElement,lastElement) 5: **end procedure**

### Appendix A.6. Sum of Euclidean Distances

Algorithm A6 presents the RelativeDistance procedure to calculate the total path length along the point sequence of a list. The list inputList contains co-ordinates and initialises a total Distance accumulator to zero. It then iterates through the list, processing each pair of consecutive points. For each adjacent pair (e.g., point *i* and point i+1), it calculates the Euclidean distance between them and adds this value to the current total stored in Distance. Once the iteration of all consecutive pairs is complete, the procedure returns the final total of Distance, which represents the sum of the lengths of all segments connecting adjacent points in the input list.

**Algorithm A6** Sum of Euclidean distances between consecutive elements in a list
1: **procedure** RelativeDistance(inputList) 2: Distance←0 3: **for** i←1 **to** length(inputList)−1 **do** 4: NewDistance← EuclideanDistance(inputList[i], inputList[i+1]) 5: Distance←NewDistance 6: **end for** 7: **return** Distance 8: **end procedure**

### Appendix A.7. Gaze Fixation Extractor

Algorithm A7 is designed to compute the duration of each distinct fixation event from a given sequence of fixation identifiers (fixations). It initialises an empty list, gaze_fixation, to hold the results. The core logic involves identifying each unique integer fixation_id within the input data, as these represent valid fixations. For every unique integer ID found, the algorithm counts its total occurrences throughout the entire fixations sequence. This count corresponds to the duration of that specific fixation, measured in the number of gaze data points it encompasses. Each computed duration is then added to the gaze_fixation list. Finally, the algorithm returns the gaze_fixation list, containing the durations calculated for all unique fixation events present in the input data.

**Algorithm A7** GazeFixationExtractor
1: gaze_fixation←emptylist() 2: **for** fixation_id in fixations **do** 3: **if** fixation_id is integer **then** 4: gaze_fixation.append(count(fixation_id in fixations)) 5: **end if** 6: **end for** 7: **return** gaze_fixation

### Appendix A.8. Gaze Movement Extractor

Algorithm A8 aims to quantify the duration of non-fixation events (movements) using identifiers present in the fixations data sequence. It initialises an empty list called movement_list and then processes the input by identifying unique non-integer fixation_id values; each distinct non-integer ID is treated as representing a specific movement event. For every unique non-integer ID discovered, the procedure counts its total frequency throughout the entire fixations sequence. This frequency corresponds to the duration of that specific movement event, measured in the number of gaze points it comprises. Each computed duration is appended to the movement_list. Finally, the algorithm returns this list, containing the calculated durations for all unique non-fixation (movement) events identified within the input data.

**Algorithm A8** GazeMovementExtractor
1: movement_list←emptylist() 2: **for** fixation_id in fixations **do** 3: **if** fixation_id is not integer **then** 4: movement_list.append(count(fixation_id in fixations)) 5: **end if** 6: **end for** 7: **return** movement_list

### Appendix A.9. Adding on ROI

Algorithm A9 describes the procedure for adding and populating a boolean column on_surface within a target data table (table). This flag indicates whether a row’s timestamp corresponds to a period, based on eye tracking data, in which the person was looking inside or outside a target space. The algorithm first iterates through each row of the target table. For each row, it checks if its timestamp falls within any time interval defined by a start_timestamp and end_timestamp pair in a separate fixations table. If the timestamp lies within any fixation interval, the on_surface flag for that row is set to True. If a row’s timestamp does not match any fixation interval, a subsequent check is performed against timestamps listed in a distinct gaze data table. If the row’s timestamp exactly matches any timestamp present in the gaze table, its on_surface flag is also set to True. The algorithm returns the modified table containing the completed on_surface column.

### Appendix A.10. Process Blink

Algorithm A10 describes the procedure for classifying blink events in relation to the region of interest (ROI), utilising corresponding gaze data that include the time stamp and the information about if the gaze was inside or outside the ROI. The inputs are a blink dataset, containing start and end timestamps for each blink, and a gaze_mark dataset which includes gaze timestamps along with a boolean status indicating if the gaze was detected ‘on surface’. For every blink event listed in the blink data, the algorithm first identifies the gaze point in gaze_mark that is closest in time to the blink’s start timestamp. It then checks the ‘on surface’ status associated with this specific gaze point. Similarly, it finds the gaze point closest in time to the blink’s end timestamp and checks its ‘on surface’ status. Based on the combination of these two status checks (i.e., whether the gaze immediately preceding and succeeding the blink was on the surface), the algorithm categorises the blink event as occurring entirely on the surface (‘all’), starting on but ending off (‘start’), starting off but ending on (‘end’), or occurring entirely off the surface (‘no’). This classification is then added to the corresponding entry in the blink dataset.

**Algorithm A9** Adding on ROI
1: **for** table.timestamp in table.rows **do** 2: **for** fixation.start_timestamp,fixation.end_timestamp in fixations.table **do** 3: **if** (table.timestamp ≥ fixation.start_timestamp) and (table.timestamp ≤ fixation.end_timestamp) **then** 4: table.on_surface←True 5: **break** 6: **end if** 7: **end for** 8: **end for** 9: **for** table.timestamp in table.table **do** 10: **if** table.on_surface==False **then** 11: **for** gaze.timestamp in gaze.table **do** 12: **if** table.timestamp==gaze.timestamp **then** 13: table.on_surface←True 14: **break** 15: **end if** 16: **end for** 17: **end if** 18: **end for** 19: **return** table

**Algorithm A10** Process Blink
1: fixations_copy← Copy of fixations_mark 2: Filter fixations_copy to include rows where ‘fixation detected on surface’ is True 3: Reset the index of fixations_copy and drop the old index 4: **for** each row in blink DataFrame **do** 5: **for** each row in gaze_mark DataFrame **do** 6: Set target1 as the ‘start timestamp [ns]’ value from the current row in blink 7: Set target2 as the ‘end timestamp [ns]’ value from the current row in blink 8: Calculate the absolute difference between ‘timestamp [ns]’ in gaze_mark and target1, assign it to diff_target1 9: Calculate the absolute difference between ‘timestamp [ns]’ in gaze_mark and target2, assign it to diff_target2 10: Find the index of the minimum value in diff_target1 and assign it to min_idx_1 11: Find the minimum value in diff_target1 and assign it to closest_value_1 12: Find the index of the minimum value in diff_target2 and assign it to min_idx_2 13: Find the minimum value in diff_target2 and assign it to closest_value_2 14: Retrieve the ‘gaze detected on surface’ value from gaze_mark at index min_idx_1 and assign it to start 15: Retrieve the ‘gaze detected on surface’ value from gaze_mark at index min_idx_2 and assign it to end 16: **if** start is True and end is True **then** 17: Set ‘on surface’ in the current row of blink to ‘all’ 18: **else if** start is True and end is False **then** 19: Set ‘on surface’ in the current row of blink to ‘start’ 20: **else if** start is False and end is True **then** 21: Set ‘on surface’ in the current row of blink to ‘end’ 22: **else if** start is False and end is False **then** 23: Set ‘on surface’ in the current row of blink to ‘no’ 24: **end if** 25: **end for** 26: **end for**
